# Optimization of Grinding Parameters of Tool Steel by the Soft Computing Technique

**DOI:** 10.1155/2022/3042131

**Published:** 2022-12-12

**Authors:** Omid Hatami, Daniyal Sayadi, Milad Razbin, Hamed Adibi

**Affiliations:** ^1^Department of Mechanical Engineering, Amirkabir University of Technology, Tehran, Iran; ^2^Department of Textile Engineering, Amirkabir University of Technology, Tehran, Iran; ^3^School of Engineering, Macquarie University, Sydney 2109, NSW, Australia

## Abstract

Grinding is one of the most complex and accurate machining processes, and the efficiency of the grinding wheel depends significantly on its surface properties. This work aims to propose an algorithmic manner that reduces the cost and time to conduct grinding of an optimized DIN 1.2080 tool steel (SPK) using a soft computing technique to obtain the best combination of input parameters including depth of cut (20, 40, 60 *μ*m), wheel speed (15, 20, 25 m/s), feed rate (100, 300, 500 mm/s), and incidence angle (0, 30, 45 de grees) with respect to output parameters consisting of average surface roughness and specific grinding energy. According to the input parameters and their levels, an experiment using fractional factorial design of experiment (RFDOE) was designed. Later on, two parallel feed-forward backpropagation (FFBPNN) networks with similar topology made up of 4, 11, and 1 units in their input, hidden, and output layers are trained, respectively. After sensitivity analyses of networks for determination of the relative importance of input variables, a genetic algorithm (GA) adopting linear programming (LP) based on Euclidean distance is coupled to networks to seek out the best combinations of input parameters that result in minimum average surface roughness and minimum specific grinding energy. The findings revealed that RFDOE provides valid data for training FFBP networks with a total goodness value of more than 1.99 in both cases. The sensitivity analyses showed that feed rate (38.97%) and incidence angle (33.94%) contribute the most in the case of average surface roughness and specific grinding energy networks, respectively. Despite the similar surface quality based on scanning electron microscopy (SEM), the optimization resulted in an optimized condition of the depth of cut of 25.23 *μ*m, wheel speed of 15.02 mm/s, feed rate of 369.45 mm/s, and incidence angle of 44.98 de grees, which had a lower cost value (0.0146) than the optimum one (0.0953). Thus, this study highlights that RFDOE with a hybrid optimization using FFBP networks-GA/LP can effectively minimize both average surface roughness and specific grinding energy of SPK.

## 1. Introduction

The most frequent machining process used to produce a fine surface finish is grinding. For the purpose of removing material, grinding machines use abrasive wheels. Several factors that impact surface finish can be generally categorized into wheel and machining factors. Abrasive grains, grit size, bonding material, wheel construction, and wheel grade are among the characteristics of the wheel. Machining parameters include the feed rate, depth of cut, wheel speed, and dressing depth for the grinding wheel [1]. Due to chip deformation resulting in elastoplastic deformation at the primary shear zone, plastic deformation and friction at the secondary shear zone, and elastic deformation and friction at the tertiary shear zone beneath the grinding wheel's cutting surface, there are several areas where material can be removed, thanks to the grinding wheel's structure, which has numerous undefined cutting edges producing a high rate of heat [2–4]. In grinding operation, the workpiece material becomes more ductile throughout the process due to the generated heat, which increases chip adherence to the abrasive tool. Heated chips from the grinding zone could have the propensity to lodge the pores of the wheel if they are not entirely eliminated from the cutting zone [5]. The clogged chips reduce the wheel's ability to cut, increasing the force and temperature of the grinding process and leading to wheel chatter or workpiece thermal damage and wheel loading. Wheel loading, which is related to the dulled wheels, decreases wheel cutting power, and the ensuing excessive rubbing and plowing, which is the propensity of detached chips to stick into the pores of grinding wheels. As a result, it enhances cutting forces and temperature while reducing wheel lifespan. In other words, the cutting forces are increased in addition to the heat flow [6–9]. This kind of damage can be reduced by utilizing flood coolant in the grinding zone. Because of the cooling action of the flood coolant, the grinding fluids eliminate part of the heat from the workpiece-wheel contact [10]. Some recent works examined adding another auxiliary nozzle in the grinding zone in order to improve efficiency. With this regard, using four compressed air nozzle jets at various angles, comprising 0, 30, 60, and 90 de grees, was conducted by Lopes et al. They found that employing flood coolant, followed by minimum quantity lubrication (MQL) with a compressed air jet at a 30 de grees angle to the wheel surface, is the most efficient method for cleaning the grinding wheel, lowering diametrical wheel wear, and surface roughness [11]. In the following, Hatami Farzaneh et al. used an auxiliary compressed air jet system to investigate the effect of the incidence angle at various angles to clean the wheel surface and reduce wheel loading. The results showed that an incidence angle of 45 de grees produced the best results in terms of surface roughness, diametrical grinding wheel wear, *G*-ratio, tangential forces, specifi;c energy, and wheel loading analysis [7].

In order to engineer the grinding process of different materials through manipulating effective parameters, methods relying on experimental data including support vector machines (SVMs), feed-forward backpropagation neural networks (FFBPNNs), fuzzy logic (FL), and neuro-fuzzy networks (NFNs) were employed for establishing a relationship to map from input variables to output variables [12, 13]. Moreover, researchers utilized different methods of optimization such as the golden section search (GSS) method, sequential quadratic programming (SQP) method, genetic algorithms (GAs), particle swarm optimization (PSO), ant colony optimization (ACO), and gray wolf optimizer (GWO), composite desirability function (CDF), and self-learning batch-to-batch optimization (SLBBO) method to seek out the best combination of input variables to reach a desirable value of output parameters [14–16].

In the following, we will review works related to the abovementioned topics. With regard to regression-based modeling, Savas and Ozay minimized the surface roughness in the process of tangential turn-milling of SAE 1050 steel by a regression-based model coupled with a genetic algorithm [17]. Bouacha et al. carried out a tentative study on the effect of cutting speed, feed rate, and depth of cut on surface roughness and cutting forces of hard turning with the cubic boron nitrides (CBN) tool of AISI 52100 bearing steel. Through designing an experiment based on the full factorial design of the experiment (FFDOE), the response parameters were modeled by response surface methodology (RSM). Then, a comparison between the optimization performance of RSM and CDF was conducted. The analogy depicted that RSM eventuates optimized conditions [18]. Elbah et al. designed an experiment based on the FFDOE to assay the effect of depth of cut, feed rate, and cutting speed on the surface roughness of the AISI 4140 steel using two inserts including CC6050WH and CC6050. Furthermore, modeling and optimization by RSM were conducted, and an optimized condition was found [19]. In another work, Bouacha et al. investigated the effect of process parameters consisting of cutting speed, feed rate, depth of cut, and cutting time on response parameters such as tool wear, surface roughness, cutting forces, and metal volume removed of hard turning of AISI 52100 bearing steel with the CBN tool. During their study, Taguchi design of the experiment (TDOE) was carried out. After modeling different responses using RSM, the optimization performance of two approaches including CDF and GA was compared. Their funding highlighted that GA is capable of resulting in better-optimized conditions than DF [20]. Qasim et al. simulated various conditions of machining AISI 1045 steel using ABAQUS software based on the TDOE. They considered cutting speed, feed rate, depth of cut, and rake angle in the orthogonal cutting process as affecting variables on cutting forces and temperature. The optimism condition was determined with regard to statistical calculations [21]. Venkatesan et al. designed an experiment using a central composite design of experiment (CCDOE) with respect to independent variables including cutting speed, feed rate, laser power, and approach angle of the laser beam axis to the tool. During their study, surface temperature and heat-affected depth of Inconel 718 alloy were modeled by RSM. According to the results, coefficients of determination of 0.96 and 0.94 were achieved for surface temperature and heat-affected depth, respectively [22]. Paturi et al. employed TDOE to investigate the effect of parameters consisting of cutting speed, feed rate, and depth of cut on surface roughness and the S/N ratio of turning of Inconel 718. During their study, multiple linear regression models were developed, and optimal statistical condition was determined [23]. Khan et al. adopted a CCDOE to perform multiobjective optimization of surface grinding of AISI D2 steel by the Gray–Taguchi method using depth of cut, table speed, cutting speed, and minimum quantity lubrication flow rate as input variables. The output variables were surface quality, surface temperature, and normal force. The objective functions were based on RSM. There were desirable results through the proposed optimization procedure [24].

Turing to ANN-based modeling, Davim et al. trained an FFBPNN with two outputs including average roughness and maximum peak-to-valley height of steel 9SMnPb28k (DIN) feeding feed rate, cutting speed, and depth of cut as effecting process parameters. To obtain data, the FFDOE was selected. A high value of the coefficient of determination (COD) was acquired in both responses [25]. Muthukrishnan and Davim applied the FFDOE considering cutting speed, feed rate, and depth of cut as effecting factors on the surface roughness of Al-SiC (20 p). Then, a model based on the FFBPNN was constructed. During the verification of the model, the maximum percentage of prediction error was 4.78% [26]. Çaydaş and Ekici compared the predictability of three different types of SVMs tools such as least square SVM (LS-SVM), spider SVM, and SVM-KM and an FFBPNN through a study to predict the surface roughness of AISI 304 austenitic stainless steel under CNC operation. The input parameters of the models were cutting speed, feed rate, and depth of cut. In order to acquire the data, a three-level FFDOE was employed. Unlike most studies, all SVMs models had better performance than FFBPNN [27]. Farahnakian et al. presented an algorithmic method based on FFBPNN as a predictive model and PSO as an optimizer for the personalization of polyamide-6/nanoclay (PA6/NC) nanocomposites products using the milling process. Under changing parameters such as spindle speed, feed rate, and nanoclay (NC) content, the cutting forces and surface roughness were minimized [28]. Davim tried to optimize surface roughness, tool wear, and power required through the manipulation of parameters such as cutting speed, feed rate, and cutting time taken from another study [29]. They integrated GA into four different techniques of modeling such as multiple linear regression analysis, response surface methodology, SVM, and FFBPNN. Overall, SVM-GA outperforms all three optimization approaches [30]. Gopan et al. reduced the surface roughness of high carbon, high chromium (HCHCR) steel by a hybrid modeling made up of FFBPNN-GA through alteration of variables including wheel speed, depth of cut, and feed rate [31]. With regard to the finish turning of AISI 4140 hardened steel with the mixed ceramic tool, Meddour et al. in addition to scrutinizing two different optimizers including the DF and the nondominated sorting genetic algorithm (NSGA-II), studied the predictability of two various methods consisting of FFBPNN and RSM to predict the cutting forces and surface roughness. The experiment was designed based on CCDOE where cutting speed, depth of cut, feed rate, and tool nose radius were affecting parameters. The finding indicated that FFBPNN-NSGA-II can result in the most desirable condition [32]. Deshpande et al. took cutting speed, feed rate, and uncut chip thickness into account to perform an experiment based on CCDOE to train two different FFBPNN for cryogenically treated and untreated Inconel 718. Moreover, regression modeling also was conducted. The analyses of model predictability demonstrated that FFBPNN with a COD of more than 0.98 is dominant over the regression method [33]. Badiger et al. used FFDOE in two series of work to investigate the effect of cutting speed, feed rate, and depth of cut on cutting forces and surface roughness. To optimize the machining condition of MDN431 with two different coatings on cutting tools including TiN/AlN and AL/Fe, FFBPNN and PSO were adopted for modeling and multiobjective optimization, respectively. They also used regression for modeling, but the results were not satisfying. Based on the optimization outcome, FFBPNN-PSO was able not only to reduce the surface roughness but also resulted in a lower cutting force [3, 34]. Kara et al. built a model based on the FFBPNN feeding cutting tool, workpiece, cutting speed, depth of cut, and feed rate to forecast the surface roughness of AISI D2 cold-work tool steel. The best performance of prediction was with a COD of 0.97 and root mean square error (RMSE) of 0.07 [35]. Susac and Stan conducted an experiment using the TDOE to investigate the effect of drill diameter, spindle speed, and feed rate on roughness, circularity error, and cylindricity error during the drilling of polymethyl methacrylate. In order to develop a relationship between input and output parameters, they utilized a feed-forward backpropagation neural network consisting of two hidden layers. The results showed that the developed models have a maximum absolute mean relative error of 7% [36]. Karthik et al. incorporated the CCDOE to study the effect of spindle speed, feed rate, and depth of cut on surface roughness AISI 316 under face milling operation. Three prediction techniques were applied to establish the relationship between dependent and independent variables, including RSM, SVR, and FFBPNN. They also constructed a hybrid model made up of FFBPNN and SVR. They found that the hybrid model of FFBPNN-SVR surpasses the other models due to partial training of weight which leads to the conversion of static learning capability to dynamic capability [37]. Ayyıldız et al. modeled the surface roughness of AA6061 alloy using two different procedures including RSM and FFBPNN considering cutting speed, depth of cut, and feed rate as input variables. They deduced that RSM could result in a higher COD due to its stability and sturdiness [38]. Kechagias et al. employed FFBPNN to map from input variables including *X* distance, *Y* distance, laser speed, laser power, and distance from top to output variables consisting of surface roughness along the *X*-axis and *Y*-axis of thin thermoplastic plates. To train networks, TDOE was utilized. Their funding revealed that FFBPNN has superior predictability power [39]. Manoj et al. made a comparison between two methods including FFBPNN and RSM to estimate the cutting velocity and surface roughness of the Altemp HX based on machining parameters such as pulse on time, wire span, and servo gap voltage. Next, GA was coupled to FFBPNN for optimization purposes due to their lower error of prediction [40].

Almost all researchers focused only on the optimization of surface roughness while less consideration has been made about specific grinding energy which is a factor that makes a remarkable contribution to the grinding process. Herein, to optimize the grinding parameters such as depth of cut, wheel speed, feed rate, and incidence angle to obtain tool steel with minimum surface roughness and minimum specific grinding energy, an experiment based on the fractional factorial design of experiment (RFDOE) is designed. Then, two parallel models via an FFBPNN consisting of four, eleven, and one node in input, hidden, and output layers are developed, respectively. Furthermore, the relative importance of input variables is analyzed. Later on, a cost function is determined and optimized by a GA to find the best individual. Finally, the optimized sample was produced and compared with the optimum sample to verify the applicability of the proposed optimization algorithm.

## 2. Experimentation

### 2.1. Workpiece Material

The material employed in the reciprocal grinding tests was DIN 1.2080 tool steel (SPK), high carbon, high chromium tool steel that has outstanding resistance to wear and abrasion and is often used in a range of mechanical applications including high-performance blanking, punching dies, and plastic molds. The SPK sample purchased from (https://www.tejarataliaj.com/) with dimensions of 20 × 30 × 10 mm^3^ had a hardness of 250 HB. In order to reduce the residual stress and hardness that might have been produced during the casting and cutting process, before the grinding process, all the samples underwent a heat treatment process as given in Table 1.

The chemical composition of this material is displayed in Table 2.

### 2.2. Experimental Design

To investigate the effect of independent variables with three different levels including depth of cut, wheel speed, feed rate, and incidence angle (nozzle angle) on average surface roughness (*R*_*a*_) and specific grinding energy (*E*_*e*_), an experiment based on Table 3 using the RFDOE is designed.

The total number of samples has been determined according to the RFDOE model [16, 41] as equation (1) utilizing Design-Expert 13 software:

(1) $$\begin{array}{c} y_{ijklm}=\mu +D_{i}+V_{j}+R_{k}+A_{l}+{DV}_{ij}+{DR}_{ik}+{DA}_{il}+{VR}_{jk}+{VA}_{jl}+{RA}_{kl}+\varepsilon_{\left(ijkl\right)m}\text{,} \end{array}$$

where *μ*, *D*_*i*_, *V*_*j*_, *R*_*k*_, *A*_*l*_, and *ε*_(*ijkl*)*m*_ are the common effects for the whole experiment, *i*th level of depth of cut, *j*th level of wheel speed, *k*th level of feed rate, *l*th level of incidence angle, and random error of *m*th repetition, respectively. The two combinations of variables indicate the interaction between them. Moreover, the average surface roughness and specific grinding energy [42–46] are calculable using equations (2) and (3), respectively:

(2) $$\begin{array}{c} R_{a}=\frac{1}{L}\int_{0}^{L}\left|Y\left(x\right)dx\right|\text{,} \end{array}$$

(3) $$\begin{array}{c} E_{s}=\frac{F_{t}V}{WRD}\text{,} \end{array}$$

where *L* refers to the sampling length. In addition, *F*_*t*_, *V*, *W*, *R*, and *D* are the tangential grinding force, the cutting speed, the contact width (20 mm), the feed rate, and the depth of cut, respectively.

### 2.3. Methodologies

In this study, according to ISO 468:1982, BLOHOM Surface Grinder was employed for grinding (model HFS204). The grinding machine was outfitted with NORTON White Alumina-32A46 JVBE 268445 vitrified bond grinding wheel. During grinding, the tangential force was extracted using the dynamometer, which is manufactured by the KISTLER Company in Germany (model 92558). Input parameters are summarized in Table 4.

**Figure 1:**
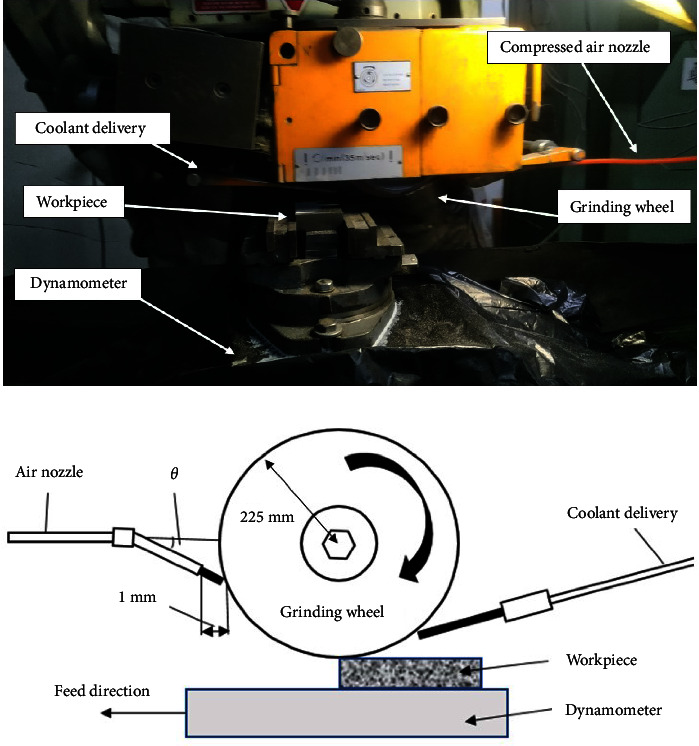
Arrangement of experimental setup. (a) During operation. (b) Schematic view.

Marsurf XR 1 surface roughness tester was employed to measure *R*_*a*_ values. According to equation (2), *R*_*a*_ is the arithmetic average of the absolute profile height values across the sampling length, which was set at 0.80 mm. Moreover, the standard wheel dimension was 225 × 37 × 51 mm^3^. The distance between the nozzle and the wheel surface was chosen to be 1 mm. Conventional cutting fluid (semisynthetic oil-based emulsion) was used as the flood coolant. The grinding wheel was dressed using a diamond single-point tool with a depth of 50 *μ*m in two passes under the speed of 100 mm/ min to obtain a wheel surface free of chips and particles after each experimental test, and its spark-out was set at 8 seconds. Additionally, the pressure of the compressed air nozzle and its dimensions was 0.70 MPa and 10 × 1 × 100 mm^3^, respectively.  Figure 1 indicates the arrangement of the experimental setup and its schematic view.

The dynamometer has been set up on the bed, and a fixture has been placed above it to hold the samples. The grinding zone contains two embedded nozzles, one for the delivery of coolant and the other for the jet of compressed air. A diamond single-point dressing mechanism has been placed on the other side of the fixture so that the time spent getting dressed can be reduced to a minimum.

## 3. Modeling and Optimization

### 3.1. Artificial Neural Network

In terms of predictions, artificial neural network-based models are considered as the most efficient ones. Among many networks, the FFBPNN is utilized to predict the output of the different systems with desirable performance. Besides, the most critical aspect during modeling with ANN is the number and validity of data obtained by performing an experiment with a particular design. Different designs including FFDOE, CCDOE, and TDOE can be taken into account to provide data for training and testing networks. In the present study, the data are obtained via the fractional factorial design of experiment (RFDOE) which considers the main and two interactions. In order to evaluate the performance of networks during training and testing steps, the same total goodness function (TGF) is considered [16, 47–49]. Besides, to enhance the performance of networks, two parallel networks with single output instead of a network with dual outputs are developed. The following functions are used for the performance evaluation of networks.

(4) $$\begin{array}{c} MSE=\frac{1}{n}\overset{n}{\underset{i=1}{\sum }}{\left(t_{i}-O_{i}\right)}^{2}\text{,}\\ R^{2}=1-\frac{\overset{n}{\underset{i=1}{\sum }}{\left(t_{i}-O_{i}\right)}^{2}}{\overset{n}{\underset{i=1}{\sum }}{\left(t_{i}-\bar{t}\right)}^{2}}\text{,}\\ GF=R^{2}+\frac{1}{e^{MSE}}\text{,}\\ \chi =\frac{n}{M}\text{,}\\ TGF=\chi_{train}{GF}_{train}+\chi_{test}{GF}_{test}\text{.} \end{array}$$

The basic components of a neural network are neurons, inputs, weights, a summation function, an activation function, and an output [35, 50]. Here, in this work, *t*_*i*_, *O*_*i*_, $\bar{t}$, and *n* are the target, output, mean values of the target, and amount of data during the testing or training step of the network, respectively. In addition, *M* is the total amount of data.  Table 5 depicts the performance of the networks using different activation functions.

Referring to the total goodness value of different activation functions, the activation function of hidden and output layers has been determined as equations (5) and (6), respectively [51]:

(5) $$\begin{array}{c} Tansig\left(n\right)=\frac{2}{\left(1+e^{-2n}\right)-1}\text{,} \end{array}$$

(6) $$\begin{array}{c} Purelin\left(n\right)=n\text{.} \end{array}$$

The learning rate and momentum value were both 0.90. All available training functions such as Levenberg–Marquardt, BFGS quasi-Newton, resilient backpropagation, scaled conjugate gradient, conjugate gradient with Powell/Beale restarts, Fletcher–Powell conjugate gradient, Polak–Ribiére conjugate gradient, one step secant, and variable learning rate backpropagation have been tried, and there was no difference in their TGF values. Thus, to improve the training process as in previous work [52], the Levenberg–Marquardt algorithm has been chosen as a training function. The number of training cycles was 1000 epochs. Both networks have a single hidden layer.  Table 6 depicts the setting of ANN-based models.

In order to specify the number of units in the hidden layer, a comparison between total goodness values was made. The abovementioned networks are developed via the ANN toolbox of MATLAB software.

### 3.2. Genetic Algorithm

A genetic algorithm is an adaptive method of optimization that is inspired by biological organisms. This procedure is based on the “survival of the fittest” that rules in natural selection, and its basic fundamentals were first affirmed by Holland [53]. By promoting the survival and reproduction of the solutions that are most likely to converge toward the optimum, these algorithms build a population of solutions and cause them to develop [54, 55]. Since it is reliable in identifying an optimal solution, which is the nearly global minimum, GA is one of the most alluring strategies for problem optimization in the numerous domains of industrial application [16, 56]. Through implementing such procedures, solutions are represented as vectors called chromosomes. During the optimization process, such chromosomes are sorted according to their either cost or fitness values which are defined by objective function or functions [14]. The process of flipping a chromosome is known as mutation. To commence optimization, the setting of the GA algorithmic toolbox of MATLAB software is considered as given in Table 7. The chromosomes change throughout a number of generations or iterations. By combining crossover and mutation, new generations are produced. In a process known as a crossover, two chromosomes are divided and then combined with each other. Later on, the chromosomal bit is flipped during a mutation. The best chromosomes are maintained while the inferior ones are eliminated after the chromosomes have been assessed using a set of fitness criteria. One chromosome is chosen as the best option for the problem after this procedure is repeated until it has the best fitness [57]. In fact, the genetic algorithm uses the criteria to determine the objective function's global minimum value and makes sure the output is the converged value. In light of this, genetic algorithms are effective tools for enhancing process parameters to identify the best-fit optimal solution from the global search based on the specified goal function to reduce average surface roughness [58]. The size of the original population, the kind of selection function, the crossover rate, and the mutation rate are the main factors that have the greatest influence on the best outcome and must be taken into account. Trial and error are used to determine the value of parameters for these criteria in order to get the desired outcome as efficiently as possible.

Regarding previous works, it can be deduced that most researchers had not considered the specific grinding energy which is the main parameter to determine the energy required for obtaining a particular average surface roughness. In this study, a multiobjective optimization is constructed based on two responses including average surface roughness and specific grinding energy. In the optimization step, both trained networks for average surface roughness and specific grinding energy are recalled, in which their value is normalized between 0.10 and 0.90. Then, a Euclidean distance [59] as equation (7) to find an individual with minimum average surface roughness and minimum specific grinding energy is applied.

(7) $$\begin{array}{c} Minimize\;:f\left(\overset{⟶}{x}\right)=\sqrt{{\left(R_{n}\left(\overset{⟶}{x}\right)-0\cdot 1\right)}^{2}+{\left(E_{n}\left(\overset{⟶}{x}\right)-0\cdot 1\right)}^{2},} \end{array}$$

(8) $$\begin{array}{c} {\overset{⟶}{x}}_{i}=\left\{D_{i}.V_{i}.R_{i}.A_{i}\right\}\;\&\;i=1.\ldots .m\text{,} \end{array}$$

where $\overset{⟶}{x}$, *R*_*n*_, and *E*_*n*_ are the vectors that store input parameters, the normalized average surface roughness, and the normalized specific grinding energy, respectively. In addition, *D*, *V*, *R*, and *A* refer to the depth of cut, wheel speed, feed rate, and incidence angle, respectively. The upper and lower boundaries of input parameters are as follows:

(9) $$\begin{array}{c} 20\leq D\leq 60\text{,}\\ 15\leq V\leq 25\text{,}\\ 100\leq R\leq 500\text{,}\\ 0\leq A\leq 45\text{.} \end{array}$$

**Figure 2:**
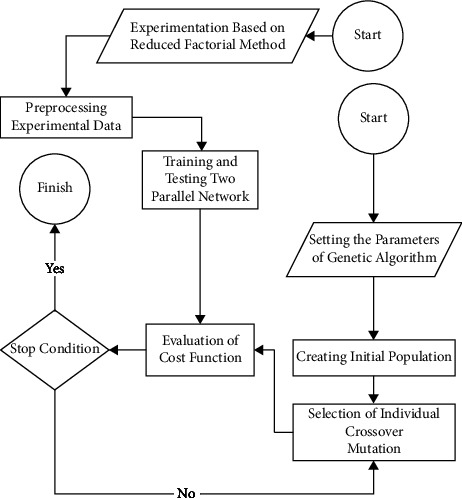
Optimization diagram of the hybrid model.

The schematic illustration of the experimentation, modeling, and optimization process is highlighted in Figure 2.

According to Figure 2, the data are normalized after obtaining them from an experiment based on the RFDOE. Next, ANN-based models are trained and tested via dataset. Later on, the GA operator is utilized to find an individual that can result in minimum average surface roughness and specific grinding energy.

## 4. Results and Discussion

### 4.1. Statistical Results

The results of the investigation on average surface roughness and specific grinding energy for 60 samples under alteration of the depth of cut, wheel speed, feed rate, and the incidence angle with a 95% confidence level are summarized in Table 8. All estimations are based on three repetitions.

Using equation (10), the raw data obtained through experimentation are normalized for the modeling step between 0.10 and 0.90 to avoid any quantitative effect [16, 60]:

(10) $$\begin{array}{c} Y_{n}=0\cdot 8\left(\frac{Y-min\;\left(Y\right)}{max\;\left(Y\right)-min\;\left(Y\right)}\right)+0\cdot 1\text{,} \end{array}$$

where *Y* and *Y*_*n*_ are the actual and normalized values of variables, respectively. Besides, to train and then test models, the dataset randomly was split into a ratio of 54 : 6.

**Figure 3:**
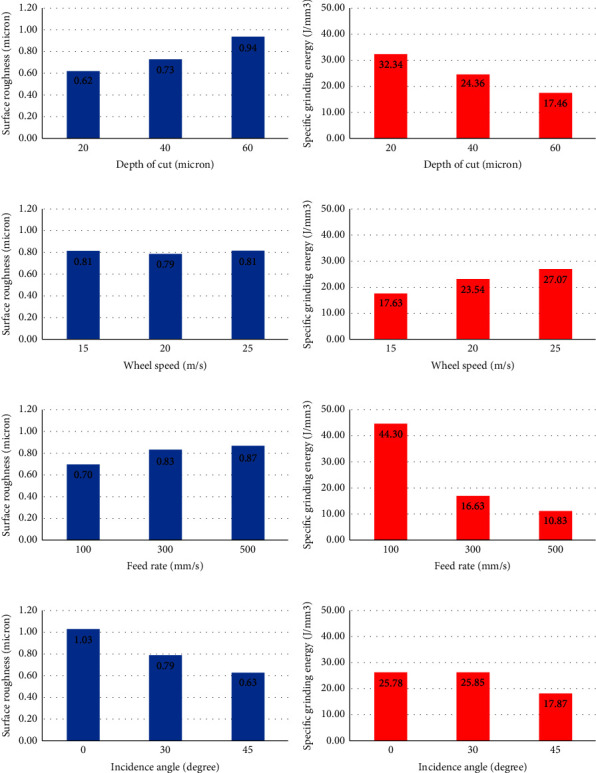
Effect of different cutting parameters on average surface roughness and specific grinding energy. (a), (b) Depth of cut. (c), (d) Wheel speed. (e), (f) Feed rate. (g), (h) Incidence angle.

Regarding the information provided in Table 8, Figure 3 demonstrates the effect of different cutting parameters on average surface roughness and specific grinding energy. According to Figure 3(a), when the depth of cut was increased from 20 to 40 *μ*m, average surface roughness rose from 0.62 to 0.92 *μ*m, respectively. Meanwhile, the reverse effect can be found in Figure 3(b) regarding specific grinding energy. With an increase in depth of cut from 20 to 40 *μ*m, specific grinding energy falls from 32.34 to 17.46 J/mm^3^, respectively. In the case of wheel speed, it can be found from Figure 3(c) that there is no significant alteration in terms of average surface roughness. At the same time, specific grinding energy underwent a growth to reach a maximum value of 27.07 J/mm^3^ at a wheel speed of 25 m/s as shown in Figure 3(d). On the other side, it can be seen in Figure 3(e) that the lowest average surface roughness (0.70 *μ*m) belonged to a feed rate of 100 mm/s while the highest one (0.87 *μ*m) was a feed rate of 300 mm/s. In addition, based on Figure 3(f), a higher feed rate (100 mm/s) led to higher specific grinding energy (44.30 J/mm^3^). Finally, changing the incidence angle from 0 to 45 de grees, as depicted in Figure 3(g), resulted in a reduction of average surface roughness from 1.03 and 0.63 *μ*m, respectively. The same trend can be found in Figure 3(h) with regard to specific grinding energy. In other words, the higher the incidence angle (45 de grees), the lower the specific grinding energy (17.87 J/mm^3^) was required.

Physically speaking, it can be said that when the depth of cut, feed rate, and wheel speed is increased, the temperature at the interface witnesses an increase that leads to the elevation of average surface roughness [35, 61, 62]. In addition, the reduction of the incidence angle from 0 to 45 de grees is investigated, and it is shown that when the angle of the nozzle is 45 de grees, a large amount of fluid is carried to interface regions which enhances the lubrication and reduces the grinding zone temperature. As a result, lower average surface roughness is achieved [62].

In terms of specific grinding energy, it is shown that a higher depth of cut causes lower specific grinding energy due to plowing and rubbing actions. In fact, the girt sharpness and grinding force are the driving forces behind the reduction of the energy [62, 63]. Through rising wheel speed, the specific grinding energy goes up due to the growing cutting force. Hence, specific grinding energy ascents [64, 65]. Moreover, a high feed rate provides a condition in which that more volume of material is removed due to higher penetration of grits. Therefore, specific grinding energy falls [66]. Similar to roughness, when the nozzle angle is 45, an optimum value of specific grinding energy is obtained due to better cleaning and cooling and lower grinding zone temperature [7, 62].

### 4.2. Developed Objective Functions

**Figure 4:**
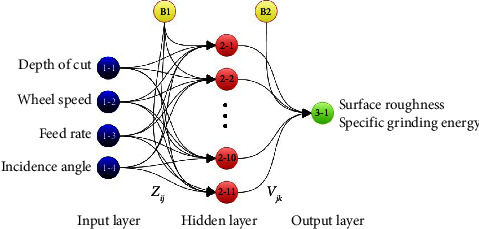
Architecture of developed neural network models.

A hidden layer, an output layer, and an input layer are frequently used to model artificial neural networks. The input layer regulates the process variables for feed rate, wheel speed, and depth of cut. The hidden layer was populated with the number of neurons required to increase the output value's accuracy. When employing a multilayer feed-forward network to solve real-world issues, one of the most crucial factors to take into account is the size of the hidden layers [67, 68]. The best network is chosen based on the total goodness value. The architecture of developed ANN-based models for average surface roughness and specific grinding energy is shown in Figure 4. Such architecture is an optimum one that can result in the highest total goodness value in both cases. Besides, it is worth mentioning that 300 runs for each topology have been taken into account due to their stochastic nature and the weight and bias values of the best-performing one are reported.

**Figure 5:**
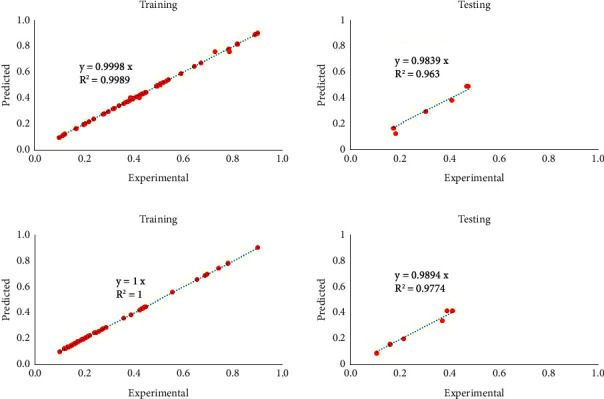
Performance of the ANN-based models during prediction at training and testing steps. (a) Average surface roughness. (b) Specific grinding energy.

Figure 5 indicates the performance of ANN-based models including average surface roughness and specific grinding energy during training and testing steps.

It can be found from Figure 5 that the capability of developed networks during both training and testing is desirable with the remarkable determination of coefficient (∼1). The training process of average surface roughness and specific grinding energy finished after 128 and 296 epochs, respectively. However, there are a few negligible settlement regions during the testing steps. Overall, ANN-based models had an excellent capability to result in a high value of TGV.  Table 9 quantitatively outlines the performance of the ANN-based models.

According to Table 9, it can be said that both trained networks have more than a 1.99 total goodness value, which demonstrates their high predictability. To construct such models as two objective functions, their bias and weight values are extracted and summarized in Tables 10 and 11.

The next phase of modeling is to analyze the sensitivity of the developed ANN-based models. Such analysis determines the impact of each input unit on the output of the network. Equation (11) will be utilized to assess the sensitivity analysis of different input variables ( *I*_*i*_) on the output of the network [69]:

(11) $$\begin{array}{c} {\;I}_{i}\left(\%\right)=\frac{\overset{n_{H}}{\underset{j=1}{\sum }}\left(\left|Z_{ij}\right|\left|V_{j}\right|/\overset{n_{I}}{\underset{l=1}{\sum }}\left|Z_{lj}\right|\right)}{\overset{n_{I}}{\underset{i=1}{\sum }}\left(\overset{n_{H}}{\underset{j=1}{\sum }}\left(\left|Z_{ij}\right|\left|V_{j}\right|/\overset{n_{I}}{\underset{l=1}{\sum }}\left|Z_{lj}\right|\right)\right)}\times 100\text{.} \end{array}$$

**Figure 6:**
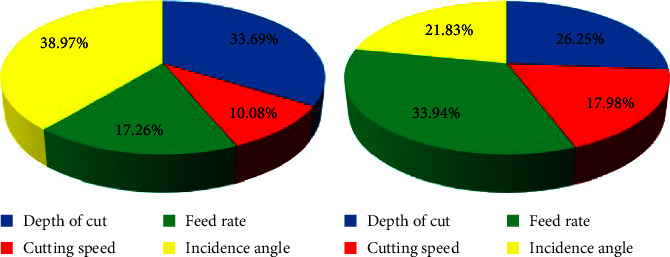
Relative importance of each input variables. (a) Average surface roughness. (b) Specific grinding energy.

According to equation (11), when the value of a specific input variable is high, it will make more contributions to the output of the network. The results of the calculation are depicted in Figure 6.

With regard to Figure 6, it can be realized that the most percentage of average surface roughness and specific grinding energy belong to incidence angle (38.97%) and feed rate (33.94%), respectively, while cutting speed (10.08%–17.98%) has the least percentage in both cases. Turing to feed rate in the case of average surface roughness (17.26%), its percentage is almost half the specific grinding energy one. On the contrary, the incidence angle in the case of specific grinding energy (21.83%) is approximately half the percentage of average surface roughness one. Moreover, the depth of cute contributes 33.69% and 26.25% with respect to average surface roughness and specific grinding energy, respectively.

### 4.3. Results of Optimization

**Figure 7:**
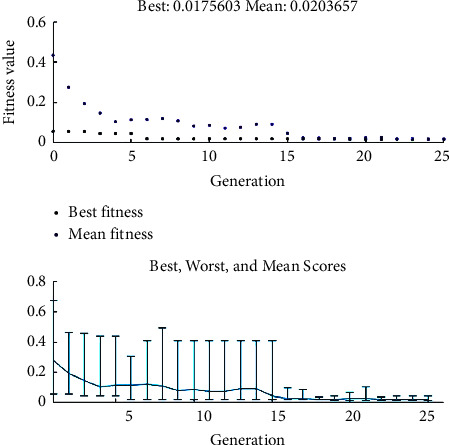
Optimization performance of GA.

When optimization is commenced, GA starts ranking individuals according to their cost value to reach out to an individual with the minimum possible cost value regarding input variables. In this study, the cost value of an individual is determined in a way that the individual with the lowest Euclidean distance to minimum average surface roughness and minimum specific grinding energy will be chosen as the best one.  Figure 7 indicates the performance of GA during optimization.

It is worth pointing out that Figure 7 is the best-performing result of 10 runs due to the stochastic nature of GA. As it can be found, the best cost value reduces during the advancement of GA until it reaches 0.0176 value over 25 generations with an initial population size of 25. Moreover, the diversity of individuals is good enough to avoid reaching local minima as a result of applying a mutation fraction of 0.50.  Table 12 compares the cost value of the optimum samples and optimized the ones considering the value of corresponding input variables and GA parameters.

**Figure 8:**
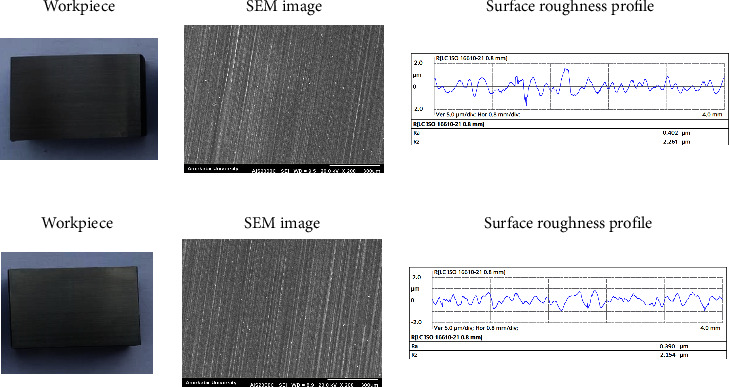
Workpiece material, SEM image, and roughness profile of different samples. (a) Optimum sample (depth of cut 20 *μ*m, wheel speed 15 m/s, feed rate 300 mm/s, incidence angle 45°). (b) Optimized sample (depth of cut 25.23 *μ*m, wheel speed 15.02 m/s, feed rate 369.45 mm/s, incidence angle 44.98°).

With regard to Table 12, it can be realized that when the population size increases, the cost value decreases. In addition, the higher the mutation faction value, the lower the cost value will be achieved. Thus, the simulated sample 10 with the highest population size and the most mutation fraction has the lowest cost value than the optimum one and other simulated ones. Regarding Table 12, the average surface roughness and specific grinding energy were reduced by 1.0308 and 2.7662 times in comparison with the optimum sample, respectively. Thus, the cost value declined from 0.0953 to 0.0146. Such reduction is the result of increasing the depth of cut from 20 to 25.23 *μ*m, keeping wheel speed and incidence angle almost constant and rising the feed rate from 300 to 369.45 mm/s. Based on the results, both depth of cut and feed rate has a direct effect on average surface roughness while an indirect effect can be found in the case of specific grinding energy. The point is that the constructed cost function (equation (7)) acts as a trade-off function between average surface roughness and specific grinding energy to find an optimized condition. To verify the result of optimization, simulated sample 10 was fabricated according to input variables.  Figure 8 depicts the workpiece, SEM image, and average surface roughness profile of optimum and optimized samples (simulated sample 10 in Table 12).

Figure 8(a) illustrates the results of the workpiece material surface of the optimum one, while Figure 8(b) belongs to the optimized one. As can be seen, the SEM images depict no damage on both surfaces, and the average surface roughness profile of the optimized one showed the lowest peaks and valleys. In fact, optimization not only improved the surface properties quantitatively but also resulted in a similar qualitative state of the surface.

## 5. Conclusion

In the following work, an experiment with a total sample of 60 using a fractional factorial design of experiment based on four independent parameters of the grinding process with three levels including the depth of cut (20, 40, 60 *μ*m), wheel speed (15, 20, 25 m/s), feed rate (100, 300, 500 mm/s), and incidence angle (0, 30, 45 de grees), and two dependent parameters of surface quality consisting of the average surface roughness and specific grinding energy was considered. Then, data were split into 90 : 10 for training and testing two parallel feed-forward backpropagation neural networks with similar inputs but different outputs. Next, the output of networks was fed to a genetic algorithm to seek out an individual with a minimum value of average surface roughness and a minimum value of specific grinding energy utilizing a Euclidean cost function. Finally, the optimized sample was fabricated to verify the optimization result. The conclusions are as follows:

1. The fractional factorial design of experiment is capable of providing valid data similar to response surface methodology for a feed-forward backpropagation neural network to reach around a total goodness value of 2
2. The proposed algorithmic procedure is not only able to find better individuals (average surface roughness value of 0.39 *μ*m and specific grinding energy value of 4.79 J/mm^3^) than already one (average surface roughness value of 0.40 *μ*m and specific grinding energy value of 13.25 J/mm^3^), but it is efficient to reduce the required time and cost value for engineering the grinding process.
3. The sensitive analysis of networks indicated that incidence angle (38.97%) and feed rate (33.94%) have the most contribution to the output of average surface roughness and specific grinding energy networks, respectively
4. The scanning electron microscopy demonstrated that both optimum and optimized samples have similar surface morphology which verifies the qualitative result of optimization.

## Figures and Tables

**Table 1:** Parameters of the heat treatment process.

Process	Temperature (℃)	Time (min)
Hardening	880	60
Cooling	880-ambient	—
Tempering	150–300	180
Annealing	300-ambient	—

**Table 2:** Chemical composition of the workpiece material.

Element	Fe	Cr	C	Mn	Si	V	W
Composition (%)	83.7	12.0	2.2	0.6	0.6	0.5	0.4

**Table 3:** Independent variables and their levels.

Variable	Code	Unit	Levels		
Depth of cut	*D*	*μ*m	20	40	60
Wheel speed	*V*	m/s	15	20	25
Feed rate	*R*	mm/s	100	300	500
Incidence angle	*A*	Degree	0	30	45

**Table 4:** Input parameters of the grinding operation.

Grinding machine	BLOHOM (HFS204)
Grinding process	Pendulum (reciprocal)
Grinding wheel	White alumina-32A46 JVBE 268445
Wheel dimension (mm)	225 × 37 × 51
Dynamometer	KISTLER Company with 92558 model
Cutting speed (m/s)	15; 20; 25
Feed rate (mm/s)	100; 300; 500
Depth of cut (*μ*m)	20; 40; 60
Conventional cutting fluid	Semisynthetic vegetable oil-based emulsion
Flow rate for the conventional technique (1/ min)	17
Air pressure in the cleaning system (MPa)	0.7
Air jet nozzle dimension (mm)	10 × 1 × 100
Workpiece material	Hardened and tempered tool steel (SPK) with dimension of 20 × 30 × 10 mm^3^
Cooling/cleaning methods	Flood coolant
	Air jet with angle of 0°, 30°, 45°
Dresser	Diamond single-point
Dressing depth (*μ*m)	50 in 2 passes
Dressing speed (mm/ min)	100
Spark-out (second)	8

**Table 5:** Performance of network with different activation functions.

Run	Activation function		*TGF*	
	Hidden layer	Output layer	*R*_*a*_	*E*_*S*_
1	Purelin	Purelin	−16.3628	−19.2731
2	Purelin	Tansig	−0.4878	1.1008
3	Purelin	Logsig	−1.1652	−1.0529
4	Tansig	Purelin	1.9987	1.9998
5	Tansig	Tansig	1.9712	1.9400
6	Tansig	Logsig	0.0317	−1.5739
7	Logsig	Purelin	−1.3190	1.3295
8	Logsig	Tansig	−0.7430	1.4582
9	Logsig	Logsig	−1.3575	−1.2885

**Table 6:** Parameter settings of the ANN-based models.

Parameter	Value
Number of units in the input layer	4
Number of units in the hidden layer	11
Number of units in the output layer	1
Activation function of the hidden layer	Tan-sigmoid
Activation function of the output layer	Pure linear
Learning rate	0.90
Momentum value	0.90
Learning function	Levenberg-Marquardt
Number of training cycles (epochs)	1000

**Table 7:** Parameter settings of GA.

Parameter	Value
Population type	Double vector
Max generation	25
Creation function	Uniform
Scaling function	Rank
Selection function	Roulette
Elite count	1
Mutation function	Adapt feasible
Crossover function	Two points
Migration direction/interval	Forward/10% of population size
Migration fraction	0.90
Nonlinear constraints algorithm	Penalty
Cost limit	0

**Table 8:** Experimental design and results.

Sample	Inputs								Outputs				Dataset	
	*D*		*V*		*R*		*A*		*R*_*a*_ (*μ*m)		*E*_*s*_ (J/mm^3^)		Training	Testing
	Act. value	Nor. value	Act. value	Nor. value	Act. value	Nor. value	Act. value	Nor. value	Act. value	Nor. value	Act. value	Norm. value	+	−
1	60	0.90	15.00	0.10	500.00	0.90	0.00	0.10	1.31 ± 0.02	0.82	7.75 ± 0.14	0.13	+	−
2	60	0.90	20.00	0.50	500.00	0.90	30.00	0.63	0.94 ± 0.03	0.53	7.87 ± 0.60	0.13	+	−
3	60	0.90	15.00	0.10	100.00	0.10	0.00	0.10	1.01 ± 0.02	0.59	31.25 ± 0.32	0.39	+	−
4	60	0.90	15.00	0.10	300.00	0.50	45.00	0.90	0.75 ± 0.01	0.39	8.00 ± 0.81	0.14	+	−
5	60.00	0.90	20.00	0.50	500.00	0.90	45.00	0.90	0.79 ± 0.03	0.42	6.93 ± 0.84	0.12	+	−
6	60.00	0.90	20.00	0.50	100.00	0.10	45.00	0.90	0.64 ± 0.01	0.30	31.67 ± 0.51	0.39	−	+
7	60.00	0.90	20.00	0.50	300.00	0.50	0.00	0.10	1.26 ± 0.01	0.78	15.33 ± 0.23	0.21	+	−
8	60.00	0.90	15.00	0.10	300.00	0.50	30.00	0.63	0.85 ± 0.03	0.47	10.17 ± 0.31	0.16	−	+
9	60.00	0.90	25.00	0.90	500.00	0.90	0.00	0.10	1.42 ± 0.02	0.90	13.75 ± 0.75	0.20	+	−
10	60.00	0.90	15.00	0.10	100.00	0.10	30.00	0.63	0.80 ± 0.04	0.43	28.50 ± 0.17	0.36	+	−
11	60.00	0.90	25.00	0.90	100.00	0.10	45.00	0.90	0.61 ± 0.04	0.28	36.25 ± 0.56	0.44	+	−
12	60.00	0.90	15.00	0.10	500.00	0.90	45.00	0.90	0.74 ± 0.02	0.39	4.65 ± 0.40	0.10	+	−
13	60.00	0.90	25.00	0.90	300.00	0.50	30.00	0.63	0.90 ± 0.02	0.51	15.97 ± 0.29	0.22	+	−
14	60.00	0.90	25.00	0.90	300.00	0.50	45.00	0.90	0.78 ± 0.01	0.41	13.19 ± 0.71	0.19	+	−
15	60.00	0.90	25.00	0.90	500.00	0.90	30.00	0.63	0.92 ± 0.03	0.52	11.25 ± 0.14	0.17	+	−
16	40.00	0.50	20.00	0.50	300.00	0.50	45.00	0.90	0.65 ± 0.02	0.32	12.67 ± 0.19	0.19	+	−
17	40.00	0.50	20.00	0.50	100.00	0.10	45.00	0.90	0.47 ± 0.03	0.17	33.50 ± 0.87	0.41	−	+
18	40.00	0.50	25.00	0.90	300.00	0.50	30.00	0.63	0.73 ± 0.02	0.38	20.83 ± 0.34	0.27	+	−
19	40.00	0.50	15.00	0.10	300.00	0.50	30.00	0.63	0.72 ± 0.04	0.37	13.38 ± 0.74	0.19	+	−
20	40.00	0.50	25.00	0.90	300.00	0.50	45.00	0.90	0.63 ± 0.03	0.30	18.13 ± 0.39	0.24	+	−
21	40.00	0.50	15.00	0.10	500.00	0.90	45.00	0.90	0.71 ± 0.01	0.36	6.75 ± 0.66	0.12	+	−
22	40.00	0.50	25.00	0.90	100.00	0.10	0.00	0.10	0.74 ± 0.06	0.38	64.38 ± 0.52	0.74	+	−
23	40.00	0.50	25.00	0.90	500.00	0.90	0.00	0.10	0.95 ± 0.04	0.54	14.75 ± 0.91	0.21	+	−
24	40.00	0.50	20.00	0.50	300.00	0.50	0.00	0.10	0.91 ± 0.03	0.51	19.17 ± 0.11	0.26	+	−
25	40.00	0.50	25.00	0.90	500.00	0.90	45.00	0.90	0.76 ± 0.04	0.40	11.75 ± 0.53	0.18	+	−
26	40.00	0.50	20.00	0.50	500.00	0.90	30.00	0.63	0.83 ± 0.02	0.45	11.20 ± 0.18	0.17	+	−
27	40.00	0.50	15.00	0.10	100.00	0.10	0.00	0.10	0.66 ± 0.05	0.32	36.00 ± 0.27	0.44	+	−
28	40.00	0.50	15.00	0.10	500.00	0.90	0.00	0.10	0.92 ± 0.03	0.52	9.00 ± 0.80	0.15	+	−
29	40.00	0.50	25.00	0.90	100.00	0.10	30.00	0.63	0.69 ± 0.03	0.34	59.38 ± 0.22	0.69	+	−
30	40.00	0.50	15.00	0.10	100.00	0.10	45.00	0.90	0.53 ± 0.02	0.22	34.50 ± 0.61	0.42	+	−
31	60.00	0.90	20.00	0.50	300.00	0.50	30.00	0.63	0.89 ± 0.01	0.49	11.44 ± 0.52	0.17	+	−
32	60.00	0.90	15.00	0.10	100.00	0.10	45.00	0.90	0.60 ± 0.05	0.28	22.00 ± 0.59	0.29	+	−
33	60.00	0.90	15.00	0.10	300.00	0.50	0.00	0.10	1.20 ± 0.02	0.73	10.50 ± 0.41	0.16	+	−
34	60.00	0.90	25.00	0.90	100.00	0.10	0.00	0.10	1.12 ± 0.01	0.67	47.08 ± 0.76	0.56	+	−
35	60.00	0.90	20.00	0.50	100.00	0.10	30.00	0.63	0.81 ± 0.02	0.44	36.00 ± 0.14	0.44	+	−
36	60.00	0.90	20.00	0.50	500.00	0.90	0.00	0.10	1.40 ± 0.04	0.89	9.47 ± 0.34	0.15	+	−
37	60.00	0.90	25.00	0.90	500.00	0.90	45.00	0.90	0.73 ± 0.02	0.38	7.75 ± 0.83	0.13	+	−
38	60.00	0.90	15.00	0.10	500.00	0.90	30.00	0.63	0.86 ± 0.05	0.48	5.10 ± 0.99	0.10	−	+
39	60.00	0.90	20.00	0.50	500.00	0.90	45.00	0.90	0.75 ± 0.04	0.39	6.33 ± 0.43	0.12	+	−
40	60.00	0.90	20.00	0.50	100.00	0.10	0.00	0.10	1.08 ± 0.05	0.65	37.00 ± 0.44	0.45	+	−
41	60.00	0.90	25.00	0.90	300.00	0.50	45.00	0.90	0.78 ± 0.01	0.41	13.06 ± 0.11	0.19	+	−
42	60.00	0.90	20.00	0.50	300.00	0.50	45.00	0.90	0.76 ± 0.03	0.40	10.89 ± 0.67	0.17	+	−
43	60.00	0.90	15.00	0.10	300.00	0.50	0.00	0.10	1.27 ± 0.08	0.79	10.08 ± 0.50	0.16	+	−
44	60.00	0.90	25.00	0.90	300.00	0.50	0.00	0.10	1.31 ± 0.07	0.82	17.78 ± 0.91	0.24	+	−
45	60.00	0.90	25.00	0.90	100.00	0.10	30.00	0.63	0.82 ± 0.01	0.45	36.67 ± 0.19	0.44	+	−
46	20.00	0.10	25.00	0.90	100.00	0.10	45.00	0.90	0.39 ± 0.04	0.11	60.00 ± 0.56	0.70	+	−
47	20.00	0.10	20.00	0.50	500.00	0.90	0.00	0.10	0.89 ± 0.06	0.49	18.00 ± 0.65	0.24	+	−
48	20.00	0.10	25.00	0.90	300.00	0.50	0.00	0.10	0.76 ± 0.05	0.40	35.42 ± 0.57	0.43	+	−
49	20.00	0.10	20.00	0.50	300.00	0.50	30.00	0.63	0.78 ± 0.05	0.41	29.67 ± 0.71	0.37	−	+
50	20.00	0.10	15.00	0.10	500.00	0.90	0.00	0.10	0.82 ± 0.04	0.45	14.10 ± 0.33	0.20	+	−
51	20.00	0.10	25.00	0.90	300.00	0.50	30.00	0.63	0.80 ± 0.01	0.43	35.42 ± 0.12	0.43	+	−
52	20.00	0.10	15.00	0.10	500.00	0.90	30.00	0.63	0.82 ± 0.03	0.44	13.65 ± 0.35	0.20	+	−
53	20.00	0.10	20.00	0.50	300.00	0.50	45.00	0.90	0.37 ± 0.01	0.10	15.00 ± 0.86	0.21	+	−
54	20.00	0.10	15.00	0.10	100.00	0.10	30.00	0.63	0.51 ± 0.08	0.20	56.25 ± 0.24	0.66	+	−
55	20.00	0.10	25.00	0.90	500.00	0.90	45.00	0.90	0.48 ± 0.09	0.18	15.25 ± 0.91	0.21	−	+
56	20.00	0.10	20.00	0.50	100.00	0.10	0.00	0.10	0.55 ± 0.03	0.24	79.00 ± 0.53	0.90	+	−
57	20.00	0.10	25.00	0.90	500.00	0.90	30.00	0.63	0.78 ± 0.07	0.41	20.50 ± 0.44	0.27	+	−
58	20.00	0.10	20.00	0.50	100.00	0.10	30.00	0.63	0.50 ± 0.01	0.20	68.00 ± 0.59	0.78	+	−
59	20.00	0.10	15.00	0.10	300.00	0.50	45.00	0.90	0.40 ± 0.06	0.12	13.25 ± 0.33	0.19	+	−
60	20.00	0.10	20.00	0.50	500.00	0.90	45.00	0.90	0.46 ± 0.01	0.17	11.60 ± 0.68	0.17	+	−

**Table 9:** Performance of the ANN-based models during the training and testing steps.

Parameter	Average surface roughness		Specific grinding energy	
	Train	Test	Train	Test
*MSE*	0.0000	0.0008	0.0000	0.0004
*R* ^2^	0.9990	0.9977	1.0000	0.9989
*GV*	1.9989	1.9970	2.0000	1.9985

*TGV*	1.9987		1.9998	

**Table 10:** Weight and bias values of the average surface roughness ANN-based model.

	Weight												Bias
*Z*	−0.9162	−0.0385	−0.0299	1.6619								1	0.5620
	−1.6992	−2.2741	−0.5367	−0.1380									2.6699
	−2.1491	−1.7052	−1.2129	−0.6057									1.8995
	4.4976	0.3169	−1.7098	−2.1134									−1.7442
	−0.0900	−0.0115	1.5718	−1.8141									4.0282
	0.6520	1.9190	−1.8986	−0.0643									0.6006
	−2.0581	3.0260	−2.4365	−0.5920									−1.0491
	−1.9916	−1.6285	−1.3518	−1.7809									−2.3489
	3.1987	0.1407	0.0930	2.3439									−0.8984
	−1.5168	1.0201	−0.7671	−0.3318									−2.9130
	1.4699	−0.3938	2.3879	1.7243									3.0665

*V*	0.8635	−0.0268	−0.0857	0.1110	0.4282	−0.0869	−0.0904	−0.1621	0.5535	0.3913	0.2190	2	−0.5015

**Table 11:** Weight and bias values of the specific grinding energy ANN-based model.

	Weight												Bias
*Z*	0.9492	2.7481	4.2005	0.3914								1	−3.9783
	0.4519	−1.5891	1.1768	−4.1529									−1.7808
	−2.3263	−1.3850	−1.2141	3.5045									−3.3722
	−3.2428	1.9768	2.4763	0.6414									1.2428
	1.2275	−1.2303	5.6614	−0.9601									3.4885
	1.3476	0.5714	2.1003	0.7893									1.6938
	1.7272	−1.5745	−3.4630	−1.2880									1.0680
	−3.1239	−0.2552	−0.7787	2.6385									3.8419
	−3.9179	0.8403	−1.0936	0.7150									−1.4050
	0.2495	−2.3161	1.6527	−2.3044									−4.4123
	−3.2897	1.6518	0.9535	−0.8230									−2.0790

*V*	−0.1249	−0.1336	−0.2084	0.2318	−0.3398	−0.3205	0.1457	−0.2001	0.0210	−0.1028	0.1517	2	−0.2927

**Table 12:** Comparison between optimum and optimized samples.

Sample	GA parameters			Input variables				*R*_*a*_ (*μ*m)		*E*_*s*_ (J/mm^3^)		Cost value	
	P.S	C.F	M.F	*D*	*V*	*R*	*A*	Predicted	Exp.	Predicted	Exp.	Predicted	Exp.
Optimum	—	—	—	20.00	15.00	300.00	45.00	—	0.40	—	13.25	—	0.0953
1	25.00	0.90	0.10	24.62	15.00	371.20	45.00	0.41	0.42	5.51	6.21	0.0337	0.0411
2	50.00	0.90	0.10	26.29	16.98	339.45	44.97	0.40	0.40	5.13	5.51	0.0225	0.0241
3	25.00	0.80	0.20	24.50	15.23	367.95	44.75	0.40	0.41	5.12	5.72	0.0196	0.0320
4	50.00	0.80	0.20	23.49	15.00	373.55	45.00	0.39	0.40	4.86	4.92	0.0181	0.0224
5	25.00	0.70	0.30	24.85	15.00	374.00	44.97	0.40	0.41	5.07	5.32	0.0192	0.0299
6	50.00	0.70	0.30	24.75	15.13	368.55	44.93	0.39	0.39	4.98	5.06	0.0176	0.0152
7	25.00	0.60	0.40	24.79	15.00	371.10	45.00	0.38	0.38	6.39	6.30	0.0194	0.0190
8	50.00	0.60	0.40	23.91	15.14	370.20	45.00	0.40	0.41	4.82	4.90	0.0188	0.0300
9	25.00	0.50	0.50	24.84	15.08	374.70	44.96	0.39	0.40	5.00	5.20	0.0185	0.0230
**10** ^*∗*^	50.00	0.50	0.50	25.23	15.02	369.45	44.98	0.39	0.39	4.85	4.79	0.0176	0.0146

P.S, population size; C.F, crossover fraction; M.F, mutation fraction; Exp., experimental value.

## Data Availability

The data used to support the findings of this study are available from the corresponding authors upon request.

## Nomenclature

*μ*: Common effect for whole experiment
*D*: Depth of cut
*V*: Wheel speed
*R*: Feed rate
*A*: Incidence angle
*R*: Surface roughness
*L*: Sampling length
*E*: Energy
*F*: Grinding force
*W*: Contact width
MSE: Mean squared error
*R* ^2^: Coefficient of determination
GF: Goodness function
TGT: Total goodness function
*t*: Target value
*o*: Output value
*χ*: Data fraction
*n*: The normalized value
*M*: Total number of data
*Y*: Variable
*I*: Relative importance
ANN: Artificial neural network
GA: Genetic algorithm
SEM: Scanning electron microscopy
FFBP: Feed-forward backpropagation
Index: Description
*i*: Level of depth of cut
*j*: Level of wheel speed
*k*: Level of feed rate
*l*: Level of incidence angle
*m*: Repetition
*a*: Average value
*s*: Specific value
*t*: Tangential component
